# An Examination of Twitter Data to Identify Risky Sexual Practices Among Youth and Young Adults in Botswana

**DOI:** 10.3390/ijerph16040656

**Published:** 2019-02-23

**Authors:** Judith Cornelius, Anna Kennedy, Ryan Wesslen

**Affiliations:** 1 School of Nursing, University of North Carolina at Charlotte, 9201 University City Blvd, Charlotte, NC 28223, USA; 2 School of Social Work, University of North Carolina at Charlotte, 9201 University City Blvd, Charlotte, NC 28223, USA; annakenn24@gmail.com; 3 Computing and Information Systems, University of North Carolina at Charlotte, 9201 University City Blvd, Charlotte, NC 28223, USA; rwesslen@uncc.edu

**Keywords:** Botswana, Twitter, risk reduction behaviors, youth

## Abstract

Botswana has the third highest rate of HIV infection, as well as one of the highest mobile phone density rates in the world. The rate of mobile cell phone adoption has increased three-fold over the past 10 years. Due to HIV infection rates, youth and young adults are the primary target for prevention efforts. One way to improve prevention efforts is to examine how risk reduction messages are disseminated on social media platforms such as Twitter. Thus, to identify key words related to safer sex practices and HIV prevention, we examined three months of Twitter data in Botswana. 1 December 2015, was our kick off date, and we ended data collection on 29 February 2016. To gather the tweets, we searched for HIV-related terms in English and in Setswana. From the 140,240 tweets collected from 251 unique users, 576 contained HIV-related terms. A representative sample of 25 active Twitter users comprised individuals, one government site and 2 organizations. Data revealed that tweets related to HIV prevention and AIDS did not occur more frequently during the month of December when compared to January and February (t = 3.62, *p* > 0.05). There was no significant difference between the numbers of HIV related tweets that occurred from 1 December 2015 to 29 February 2016 (F = 32.1, *p* > 0.05). The tweets occurred primarily during the morning and evening hours and on Tuesdays followed by Thursdays and Fridays. The least number of tweets occurred on Sunday. The highest number of followers was associated with the Botswana government Twitter site. Twitter analytics was found to be useful in providing insight into information being tweeted regarding risky sexual behaviors.

## 1. Introduction

Rates of sexually transmitted infections (STIs) are high in Botswana, and the HIV infection rate is the third highest in the world [1]. The Republic of Botswana Ministry of Basic Education reported that Botswana youth and young adults (15 to 24 years of age) are aware of STIs, but gaps exist in knowledge relevant to modes of transmission [2,3]. As a result, there is a rapid increase in STIs among this population, beginning at age 15, making youth a priority for prevention efforts [2,3]. Botswana also has one of the world’s highest mobile cell phone density rates, exceeding that of many industrialized countries like the United States, United Kingdom and Germany, with 144 mobile phone subscriptions per 100 people for an estimated 99% of the Botswana population [4]. Botswana youth and young adults have access to social networking sites and many use Twitter, Facebook, and Whatsapp [5]. In African countries such as Botswana, the trend with social media and its impact is not well documented in the literature [5]. Over a 17-year period, Internet use in Botswana has increased by 6.057% [6]. Content from social media may be one way of reaching youth and young adults with safer sex information. An interdisciplinary research team from an American university explored Twitter data in Botswana to determine trends in the demographics, gender distribution, daily schedule, and key words related to HIV prevention and awareness of safer-sex practices.

### 1.1. Difference Between Twitter and Other Social Media Networks

While social medial networks allow for personal branding, differences exist. Compared to Facebook, people who use Twitter tweet and their followers retweet the tweeted information [7]. Facebook allows likes, friends, or favorites. Twitter has a more targeted audience and Facebook is mainstream. Twitter tweets have a shorter lifespan than a Facebook update. Speed is also a factor in that Twitter is faster in transporting messages than Facebook. So if a person wants to create an instant viral effect or provide important updates, then Twitter proves to be more effective [7]. Twitter tweets are in the public domain unless Twitter users have marked them as private. With Twitter, a person can talk to online friends interested in similar topics, making it a prime source for examining risky behaviors of a group [7].

### 1.2. The Impact of Social Media on Health Outcomes

Social media shows promise in the dissemination of mobile health information. A recent focus group with Botswana teens indicated that using social media to disseminate health promotion information about safer-sex practices is feasible and acceptable [8]. To date, social media interventions have focused on population health, [9] smoking cessation awareness [10], weight reduction [11], and behavior change among college students [12]. Specific to HIV awareness, we found three studies [13,14,15] that examined the effect of using social media to increase HIV awareness among men who have sex with men (MSM); two studies used social media to increase HIV care linkage, retention, and health outcomes [13,14]; and another study that used social media to increase HIV testing among MSM in Peru [15].

Even fewer studies have examined the use of social media to deliver safer-sex information to adolescents. We did find one study that used Facebook to deliver a sexual health intervention to youth. Study outcomes indicated that the intervention was effective in increasing condom use [16].

Meta-analyses demonstrate that computer- and internet-based interventions contribute to improved sexual health outcomes for youth at risk [17,18,19], and outcomes are as good as established, non-technology-based sexual health programs [17]. However, research on promoting healthy behaviors among youth suggests that to be effective, interventions must be tailored to the study population and culture [20,21]. To our knowledge, only two HIV media-related prevention programs have been developed for Botswana youth. In the first study, a video series called the Wise Up program raised awareness about HIV prevention [22]. In the second study, a social media online discussion forum was formed with college students enrolled in one course to change HIV behavior of the participants. The social media campaign encouraged Botswana youth to be tested for HIV, and succeeded in increasing the number who did [23]. Findings from these studies demonstrate the promise of social media in improving public health.

Healthcare providers have also embraced Twitter as a means of disseminating health information. In one study, Twitter messages expanded the reach of breast cancer awareness among individuals during breast cancer awareness month [24]. In the first study to compare social media messages about breast, prostate, and other reproductive cancers, Twitter generated more activity during cancer campaigns to reach the public when compared to Instagram [25]. In another study, pro-marijuana messages underscored the need for surveillance efforts to monitor the reach of these messages to youth [26]. On one website with 236,000 followers, Twitter messages were effective in increasing awareness of cervical cancer risks of HIV positive women during World AIDS Day [27]. In another study, a content analysis of over 2 million Twitter posts during the HINI pandemic identified the value of social media to inform populations during a pandemic [28]. However, we found only a few studies that examined the dissemination of safer-sex information using social media [29,30]. In one study, youth exposed to social media health messages were 2.69 times more likely to have used condoms at last intercourse [29]. In the other study with 900 participants, the authors concluded that further work is needed to reach and engage social media audiences [30].

Social media safer sex interventions when compared to in-person interventions show potential in reaching and engaging larger groups of people. However, before we can begin to design interventions, we need to analyze Twitter messages that occur around a focal time period of HIV prevention awareness. Therefore, the purpose of this study was to analyze a three-month sample of tweets from Botswana around the focal event of World AIDS Day, to examine characteristics of the top 25 active users, and to identify key words used for risky sexual behaviors. The long-term goal of our research will be to develop a social media risk reduction intervention for youth and young adults in Botswana.

### 1.3. Research Questions

For this study, we identified three research questions:

1. What are the demographic characteristics of Twitter users who sent tweets around the focal event of World AIDS Day?
2. What are the key words associated with risky sexual behaviors?
3. From the content analysis of tweets, what are trends in risky sexual behaviors that could be used to develop a risk reduction intervention delivered via social media?

## 2. Materials and Methods

In this exploratory quantitative descriptive study, we pulled a three-month sample (1 December 2015 to 29 February 2016) of Tweets from Botswana using Gnip’s Historical PowerTrack, an application programming interface (API) [31]. Gnip is Twitter’s API platform that offers proprietary tools to retrieve historical and streaming data. Unlike publicly available APIs that sample a percentage of available data, Gnip’s API queries all tweets using specific filtering rules. For the initial dataset, the filtering criteria included two rules based on user and tweet location within the three-month timeframe. The first rule limits the collection to tweets by Twitter users whose self-identified location was Botswana (country code = BW). The second rule was based on geolocation (point or place coordinates), requiring the tweet to be within a region around Gaborone, Botswana (the site of greater internet access in the country) the site for our future research. The original dataset was constructed in a Javascript object notation (JSON) format then converted to concurrent version systems (CSV) format for analysis.

Each tweet was classified as original. We did not analyze retweets since the original tweet would have included information about the risky behavior. Using the terms listed in Table 1, we used Twitter API to filter the general tweet-stream, to find those tweets that contained key words relevant to risky sexual behaviors. Although the official language in Botswana is English, Setswana is widely spoken, so we hired two graduate assistants to translate tweets in Setswana into English. We prepared a list of topics associated with terms associated with risky sexual behaviors and based on the first author’s research experience. To gather the HIV-related tweets, we searched for key words in English and Setswana. The total number of tweets collected was 140, 240. Using key filter HIV-related terms, we collected 576 original tweets from 251 unique users, representing 0.4% of the total tweets in a 3-month time period. Additionally, we classified Twitter users as individual or organizations. An individual was categorized as a user if they had followers and a verified Twitter account. An organization was categorized as a user if their screen name used one or more of the following key words, agency, health, government, company or firm.

### Data Analysis

After converting the data from JSON into CVS format, the data were analyzed in R Studio to identify HIV-related tweets. An initial analysis of tweets over time showed that there was an unexpected peak on World AIDS Day, 1 December 2015. We were able to calculate simple statistics since the number of tweets was less than 50,000. We tested for differences among individuals or organizations using t-tests and tested for differences among the number of tweets each month (December–February) using ANOVA. For content analysis, we used computational linguistics to automatically identify topics. We cleaned the data then used key words to analyze content in the tweets. Research assistants experienced with the Botswana culture and language, analyzed the content. Each tweet was reviewed for syntax, semantics, linguistics, and key word and key phase abstraction. Each tweet was reviewed until agreement was made with the content.

## 3. Results

### 3.1. Characteristics of the Users

A total of 140,240 tweets were collected, with 500 to 2500 tweets per day and an average of 1500 tweets in the Gaborone area. The tweets came from approximately 168 males and 83 females. Some individuals used a combination of English and Setswana in their tweets. Of the top 25 active Twitter users (*n* = 576 HIV-related tweets) including individuals and organizational Twitter sites, the number of friend counts ranged from 173 to 5001. Their followers ranged from 1121 to 43,945. The highest number of followers represented the country’s Botswana Government Twitter site (*n* = 43,495).

Individuals and organizations (Botswana Government site, Public Relations Firm and Health Department) were similar in the content of their tweets. Emphasis on HIV prevention and testing was not significantly different in the month of December when compared to January and February (t = 3.62, *p* > 0.05) and occurred primarily during the morning and evening hours and on Tuesdays, followed by Thursdays and Fridays. The least number of tweets occurred on Sunday. The number of tweets each day ranged from a minimum of 1 tweet up to 31 tweets daily (average 6.33). Twitter tenure ranged from 1.34 to 6.7 years. The users used mobile devices more frequently than desktop computers to gain access to the Twitter site. The primary mobile device used to access Twitter was the android phone followed by the web and iPhone.

### 3.2. Risky Behaviors

Words related to HIV/AIDS and testing occurred more frequently during the three weeks following World AIDS Day (December). There was no significant difference between the numbers of HIV related tweets that occurred from December (*n* = 200, January, *n* = 198) to February (*n* = 178) (F = 98.1, *p* > 0.05). No tweets pertaining to HIV testing occurred during the month of February. Table 2 shows the frequency of the 576 tweets and content analysis of the top 25 users’ commonly used words pertaining to sex or sexual behaviors in English and Setswana. The tweets more commonly had content related to HIV testing, AIDS, sexual activity, protection, risks, and drugs.

### 3.3. Use of Hashtags

Of the sample of tweets from the top 25 active Twitter users, hashtags #WorldsAIDSday or #WorldsAIDSDay prevailed. The Worlds AIDS Day hashtag had the highest frequency use of the dataset, despite only being tweeted twenty times. Of the tweets that used the hashtag, there were positive messages about reducing HIV and getting tested. One tweet said “Today is #World3Day:GETTING TO ZERO- Zero New HIV infections, Zero Discrimination, Zero AIDS Related Deaths, Whn was the last time u tested?” Another tweet said, “Oh....nd as u scream ‘Helloooooooo Dcmbrrrrrrr’ dnt frgt its #WorldAidsDay, so go get tested for HIV le lese go tlhodia (make some noise)”. Showing compassion for those already infected with HIV, one tweet said, “A friend with HIV is still a friend”. Fear of this disease was evident in one tweet, “I’m scared to have sex without a condom”, and “Kana le ga o dirisa condom (pack a condom) you are still at risk, what if it burst?”

### 3.4. Trends in Twitter Messages

We were able to translate key concepts from the Twitter data to identify if trends for risky sexual behaviors exist. Examples of how major educational elements can be used to address these trends are identified in Table 3.

## 4. Discussion

In this study, we analyzed characteristics of Twitter users in a high-risk HIV country. Consequently, we sought to characterize the top 25 active Twitter users in our sample who tweeted about safer-sex practices. Results showed that tweeting about HIV/AIDS and HIV prevention was a singular event that occurred during the first few weeks in December, fell off in January, and was virtually non-existent in the month of February. We cannot explain why Twitter was popular on Tuesday, but low Twitter use on Sunday can be explained by the fact that people in Botswana are very religious and therefore may spend their time involved with religious activities on Sunday. The high volume of Twitter activity underscores the importance of using social media as a platform for health-related discussions on topics such as HIV prevention. In addition, previous research has shown social media sites to be effective in delivering information relevant to HIV testing and substance abuse [14,15,32].

The association between Twitter messages and World AIDS Day for this study is a coincidence. We began data analysis at this time point to see how long HIV prevention messages would be tweeted. This study used Twitter as a sample representative source of data on real-life communication and with naturalistic behavior. In 2013, mandatory HIV testing became legal in Botswana [2,3]. In February, HIV testing and knowing your status messages were not tweeted. With time greater emphasis should be placed on keeping people engaged with messages about safer sex practices. We also could not infer if the tweets came from HIV positive or HIV negative individuals. Each tweet had a different meaning in relation to prevention or risky behaviors. For example, PrEP and condoms are recognized as treatment for serodiscordant couples, and condoms and additional barriers (dental dams) are recognized for non-serodiscordant couples.

The largest number of Twitter followers was from the Botswana government’s Twitter handle. In terms of tweets that contained medical advice, the government tweet provided references. This finding is significant in reference to the dissemination of reliable information on safer sex. Castillo, Mendoza and Poblete [33] noted that tweets providing explicit references to back claims (tweets) had significantly more retweets than those without. However, despite showing a preference for tweets with supported references to information, both scientifically accurate and inaccurate tweets may be retweeted, indicating that social media users may have a difficult time determining what is fact and what is fiction. This finding is timely given the current controversy surrounding the spread of fake news on Facebook and Twitter [33,34].

The number of new infections has decreased but more can be done. For example, our data did show that substance abuse (drugs and alcohol) use was frequently discussed on Twitter, along with pro-drinking messages. This finding has been corroborated by earlier studies on the prevalence of alcohol abuse in Botswana [35,36]. It has been noted that an individual in Botswana consumes in excess of 20 liters of alcohol per year [37]. The link between excessive drug and alcohol use and sexual practices is well established [36]. Botswana youth begin drinking at an average age of 15, and they begin having sex at 17.5 years [36]. The Twitter data in this study supports the popularity of tweets surrounding alcohol use. The results from this analysis indicate a need for surveillance data to monitor the kind of risk-reduction messages that are most effective for this population.

Twitter use among youth and young adults is high. This generation has unique characteristics of mobile phone and social media use that lend this group to be targeted for social media health awareness campaigns. Twitter health promotion campaigns have addressed topics of interest for this age group such as: alcohol awareness, dating violence, and cancer detection. Participants in this sample more frequently used mobile cell phones to gain access to social media sites. To expand the reach of messages, researchers need a strategic communication plan to ensure ongoing social media conversations that encourage HIV prevention, testing, and treatment.

Twitter has become an essential part of the dissemination of health promotion information, but little information is available about whether social media in a country with high rates of HIV infection has been effective in teaching safer-sex practices. Trends in data can identify key areas in which prevention efforts can be targeted in Botswana. For example, HIV knowledge, protection, safe sex negotiation, and alcohol/drugs are key topics of HIV prevention curriculums in the United States [38], but the exchange of money for sexual favors is not. This is one major cultural difference that will need to be addressed in a safe sex social media intervention in Botswana. Finally, passing the message on is one way to encourage dissemination of safe sex information, which is the primary purpose of Twitter tweets.

### Limitations

This study has several limitations. First, the demographics of followers are not actual reported data but rather inferred data based on Twitter behavior/usage. Twitter is growing in popularity, but is still behind Facebook and Pinterest in Botswana [39]. Second, we report on a three-month sample and around the focal event of World AIDS Day. The tweets may not be representative of tweets throughout the year, as many of the Twitter users may have focused on the event and may have personally known someone infected with HIV or died of AIDS. Third, this study only represents Twitter tweets available to the public. Those marked private are not included. Last, we have no way of knowing if the Twitter users or followers were sexually active and practiced risky sexual behaviors or engaged in alcohol abuse. We can only infer these things based on their tweets as to what the popular messages were at this time.

## 5. Conclusions

Despite these limitations, our study indicates the need for continued research and surveillance data on messages being broadcasted via Twitter. Twitter use has expanded exponentially, especially among youth and young adults. Twitter messages that encourage safer sex practices could be helpful in getting HIV information to youth. Twitter users were more likely to follow the government website and trust the information presented there. Twitter could become a powerful tool in the fight against HIV and AIDS as a communication tool to spread messages about safer sex practices and alcohol prevention. The data from this exploratory study could be a first step for health care providers to create initiatives that disseminate the latest HIV knowledge and increase awareness of HIV prevalence in high-risk areas. Additional research should be conducted to address who would initiate a Twitter-based safer sex intervention, what content would it involve, how can people stay engaged, what will be the dosage of tweets, and how can we assess its effectiveness?

## Figures and Tables

**Table 1:** Key filter terms.

Setswana	English
mogare wa AIDS	AIDS
go suna	kiss
Motsoko	Smoking drugs
Lepele	Genitals (penis, vagina)
Bojalwa	alcohol
Biri	beer
Motokwane	cannabis
go betelela	rape
segajaja	World AIDS Day

**Table 2:** Frequency of the 576 Tweets and content analysis of the top 25 users’ commonly used words pertaining to risky behaviors.

English	Setswana	Frequency	Terms
HIV/AIDS	Phamokate, Mogare wa HIV	47	HIV, sexually transmitted infection, death, AIDS
HIV testing	Go Testa, Go ichel	43	Health, protection, awareness, status
Sex	Thobalano, Tlhakanelo Dikobo	42	Intercourse, sexual, sex, pleasure, feel good
Condom	Sekausu, Kgothlhopo,	38	Wrap, protection, rubber, penis, contraceptive,
Kissing	Go Suna	37	Lips, touch, sign of love, passion
Sexual risks	Kkotsi ya thobalano	36	Pregnancy, infection, HIV, AIDS
Penis	Motsoko, Lepele, Bonna	35	Male, genital, sperm, erection
Alcohol	Bojalwa, Biri	32	Wine, beer, spirits,
Drugs (Cocaine, Heroin, Crack, Meth,	Diritibatsi	30	High, pleasure, inject, snort
Weed	Zolo, Dipatshe, Motokwane, Dagga	27	High, pleasure, hunger, smoke, blunts, cannabis
Vagina	Kukunas, Kkuku	25	Female, genital
Abstinence	Iphapo	24	Without, no genital contact
Protection	Go thibelela	23	Birth control, pill, reproductive health, barriers, wraps
Negotiate	Go buisana	20	Talk, safe, agreement
Immune Deficiency	Koafalo, Tlhaelo ya phemelo ya mmele	19	Disease, AIDS, HIV
Violence	Go Iwa, Go betha	16	Physical force, power, hurt
Rape	Go betelela, Thubegetsa, kgapeletso ya thobalano	15	Sexual, assault, penetration
Anal	Phatlha ya marago	14	Butt, rectum
Abuse	kgokgontsho	13	Physical, emotional, effect
Incest		11	Sex with relatives, younger person
Abortion	Tshenyegelo, Phetelelo	10	Pregnancy, terminate
Dominance	Bonna	7	Influence, power
Body Piercing	Go iphunya	5	Body parts, holes, earring, jewelry
Tattoo		2	Body art, markings
Partner	Mokapelo, Moratuwa	2	Married or engaged
Prostitution	Lebelete/Mabelete	2	Money, sex exchange
Peer Pressure	Peer pressure	1	Influence, member peer, group

**Table 3:** Twitter topics related to safe sex practices.

Trend	Key Concepts	Example of Twitter Posts
HIV knowledge—what do I know and not know	Epidemiology, signs and symptoms, testing and prevention methods. Reducing anxiety with HIV testing	“I’m worried about the HIV testing average of 63%. I’m going to test immediately after this! Acting President @OfficialMasisi @BWGovernment “Gaborone youth topping in new HIV infections... #MoH”
Proud2BSafe—Reasons why I am proud to be safe	Negotiating safer-sex communication, condom use	“Condoms are used and bought, so if a condom with a certain batch number or expiry date disappears, I got news for you—no sex.”
Blessers—How to stay safe with men or women who bless you	Being Blessed but Being Safe, Intergenerational sex, safer sex practices	“Y’all have sex with a man. He spends money on you. You call that a “blessing,” as in he’s your “blesser,” like you sleeping with Jesus? SMFH”
Alcohol Use—Can lead to unsafe sexual practices	Consequences of alcohol use and unsafe sexual behaviors	“I drink and then I have sex without a condom.”
Passing on the message—Let others know how to stay safe	How to pass along safer-sex messages	“How AIDS Advertising Has Evolved from Shock and Shame to Hope and Humor” | Adweek https://t.co/xcUsbrUvOI
